# A model for simulating Local Field Potential in the thalamus of Essential Tremor patient during deep brain stimulation

**DOI:** 10.1186/1471-2202-13-S1-P40

**Published:** 2012-07-16

**Authors:** Ishita Basu, Daniela Tuninetti, Daniel Graupe, Konstantin V Slavin

**Affiliations:** 1 Department of Electrical & Computer Engineering, University of Illinois at Chicago, USA; 2 Department of Bioengineering, University Illinois at Chicago, USA; 3 Department of Neurosurgery, University Illinois at Chicago, USA

## 

Local Field Potential (LFP) is simulated by adding the neuronal membrane potential of a group of model neurons surrounding a typical intra-operative micro-electrode used to localize the target for deep brain stimulation (DBS) in Essential Tremor (ET). Each neuronal membrane potential is modeled as an Ornstein Uhlenbeck Process (OUP) and the model parameters are extracted from real neuronal recording during DBS surgery following the method in [1].

Before any test stimulation through the macro ring of the microelectrode, the LFP is simulated according to:(1)

Where, *x* is the membrane potential of each neuron and r is the distance from the recording tip. The simulated signal, *lfp* is then low pass filtered (1-40 Hz) to produce the final signal.

With stimulation on, the LFP is simulated similarly but only over time intervals in between two stimulation pulse which is free of the stimulation voltage artifact caused by amplifier saturation. The stimulation artifact time duration is estimated as:(2)

Where, *l*(*r*) is the stimulation intensity at distance r from the recording tip and follows a similar inverse square decay as in (1), τ is the membrane time constant (an OUP model parameter) and S is the neuronal firing threshold. Thus the LFP simulated during stimulation depends on the stimulation intensity, pulse width and frequency.

The stimulation parameters used are: frequency: 160 Hz (125 samples/period), pulse width: 0.4 ms, intensity:1.5 mA

It was found that the power in the (5-12) Hz band decreases by 26.4% during stimulation. There is increased synchronization in neuronal activity of the thalamus in the theta band (4-7 Hz) for patients with ET [2]. The suppression of tremor by DBS is reflected in the reduction of power in the tremor frequency band.

Such a model , can potentially be used to find an optimal set of stimulation parameters that will produce the maximum suppression in the tremor power.

**Figure 1:**
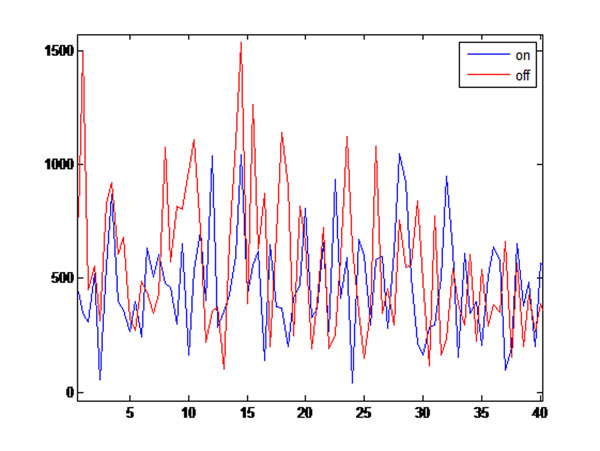
Spectrum of LFP before stimulation (in red) and during stimulation (in blue).
